# A rare presentation of thyroid malignant peripheral nerve sheath tumor in neurofibromatosis type 1 delineated by FDG PET/CT: A case report and literature review

**DOI:** 10.22038/AOJNMB.2024.76555.1539

**Published:** 2024

**Authors:** Serin Moghrabi, Nabeela Al-Hajaj, Fatimah Abu Aljaaz, Omar Jaber, Akram Al-Ibraheem

**Affiliations:** 1 Nuclear Medicine Department, King Hussein Cancer Center, Jordan; 2 Department of Pathology, King Hussein Cancer Center, Amman, Jordan

**Keywords:** Malignant peripheral nerve, sheath tumor, Neurofibromatosis type 1, Thyroid, PET/CT A B S T R A C T

## Abstract

Malignant peripheral nerve sheath tumors (MPNST) are rare, aggressive soft tissue sarcomas that arise from peripheral nerves and often present a diagnostic and therapeutic challenge. They can occur sporadically or in association with neurofibromatosis type 1 (NF1), a genetic disorder caused by mutations in the NF1 gene. This report presents the unique case of a 33-year-old male with progressive dry cough, hoarseness, and neck swelling who underwent a total thyroidectomy, revealing a high-grade malignant peripheral nerve sheath tumor invading the thyroid. FDG PET/CT led to the additional diagnosis of NF1. This case stands out due to the rarity of finding an MPNST within the thyroid and the simultaneous identification of NF1. It underscores the importance of screening MPNST patients for NF1 and vice versa, spotlighting the expanding role of FDG PET/CT in comprehensive evaluations. To our knowledge, this report presents the first case of NF1-associated MPNST with thyroid involvement worldwide.

## Introduction

 Malignant peripheral nerve sheath tumors (MPNST) are a subset of rare soft tissue sarcomas that arise from peripheral nerves (1). 

 They represent around 2% of all sarcomas, with an incidence rate of 0.001% (2, 3). Even though MPNSTs can be sporadic, they are usually connected to neurofibromatosis type 1 (NF1), a genetic disorder caused by changes in the NF1 gene on chromosome 17 (4). MPNSTs can occur in various anatomic sites, being more frequent in the lower and upper extremities, followed by the trunk, and least commonly in the head and neck region, with a prevalence of 2–9 % of all cases (5, 6). MPNSTs involving the thyroid constitute an exceedingly uncommon subset of neoplastic entities, accounting for less than 0.02% of all thyroid tumors worldwide (7). 

 MPNSTs pose a significant diagnostic challenge due to their aggressive nature and potential for eccentric metastasis, which requires a comprehensive diagnostic approach involving CT, MRI, and, recently, FDG PET/CT.

 This case is especially unique because of the malignant peripheral nerve sheath tumor (MPNST) that was found in the thyroid and the subsequent diagnosis of neurofibromatosis type 1 (NF1). This shows how these conditions interact with each other and how difficult it can be to diagnose them. To the extent of the search we have done in Medline, this report presents the first case of NF1-associated MPNST with thyroid involvement worldwide.

## Case report

 In June 2023, a 33-year-old male patient with no significant past medical or family history, presented with a chief complaint of a chronic dry cough persisting of nine months duration, showing no improvement despite using cough suppressants. The cough was associated with progressive dysphagia, hoarseness of voice, shortness of breath, and anorexia. Notably, the patient denied experiencing palpitations, tremors, thermal sensitivity disturbances, nervousness, insomnia, ocular manifestations, or alterations in bowel habits. Upon physical examination, a well-defined nodule located in the left thyroid lobe was noted. The nodule measured approximately 3-4 cm in diameter and appeared to be solid in consistency. It was non-tender to palpation and felt fixed to the surrounding thyroid tissue. Moreover, his thyroid function and other laboratory tests were unremarkable.

 A subsequent neck ultrasound revealed the presence of a substantial left inferior thyroid nodule measuring about 4.9 by 4.3 cm and extending into the retrosternal region. This nodule exhibited heterogeneity, internal vascularity, and cystic changes. There was no evidence of enlarged cervical lymph nodes.

 FNA did not show evidence of cancerous cells. However, the treating team decided that surgical intervention was necessary due to compressive symptoms and he underwent total thyroidectomy in July 2023 which revealed a 6.5 cm tumor adjacent to the left thyroid lobe. 

**Figure 1:**
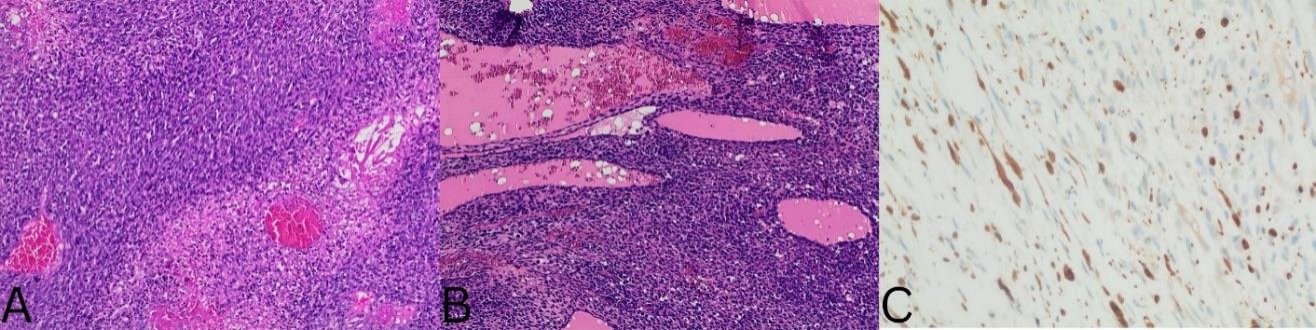
The tumor is composed of malignant spindle cells arranged in fascicles with high mitotic count and necrosis (Hematoxylin and eosin stain, 200X). **B**. The tumor involves the adjacent thyroid parenchyma (Hematoxylin and eosin stain, 200X). **C**. The tumor is focally positive for S100 protein indicative of neural differentiation, (S100 protein immunohistochemical stain. 200X)

 Histopathology reported a high-grade spindle cell sarcoma with negative inked margins and evidence of thyroid invasion. The histological and immunohistochemical features showed a malignant spindle cell tumor arranged in intersecting fascicles, which were focally positive for S100 and SOX10 and negative for pan-CK, STAT6, CK-HMW, CD34, SS18, TTF-1, SMA and PAX-8. Given the evidence of neural differentiation by immunohistochemistry, the diagnosis of MPNST was confirmed consistent with a high-grade malignant peripheral nerve sheath tumor involving thyroid parenchyma (Figure 1). 

In August 2023, the patient was referred to a cancer center for dedicated assessment and treatment. In his first appointment, he complained of pain and swelling at the site of surgery. On clinical examination, six skin lesions with a reddish to brownish color, measuring about 1 cm each, were noted on his lower back, in addition to seven café au lait spots, measuring about 2 cm, observed on his bilateral upper and lower back. These spots had been present since birth, as reported by the patient, which raised the suspicion of neurofibromatosis. A whole-body CT showed two large pleural lesions and a right sciatic foramen soft tissue lesion, with sizes ranging from 4.0 to 4.6 cm, but no suspicious focal lesion in the surgical bed. A neck MRI showed suspicious residual nodules in the lower posterior left thyroid bed between the left major neck vessels, measuring about 1.9 cm. 

**Figure 2:**
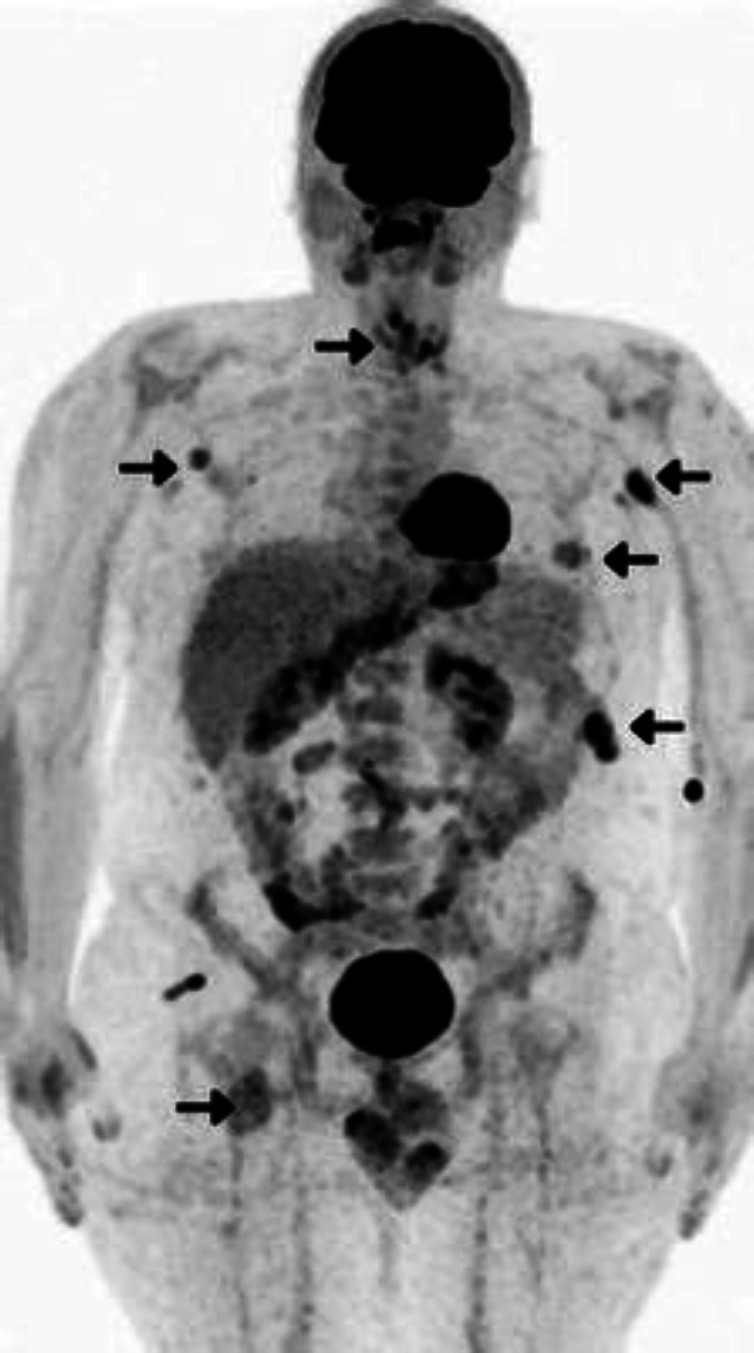
Positron emission tomography/computed tomography (^18^F-FDG PET/CT) MIP images demonstrate hypermetabolic lesions in the bilateral thyroid bed, as well as hypermetabolic two left pleural and intercostal soft tissue masses, in addition to soft tissue lesions in bilateral axillary regions and right inferior pelvis

**Figure 3:**
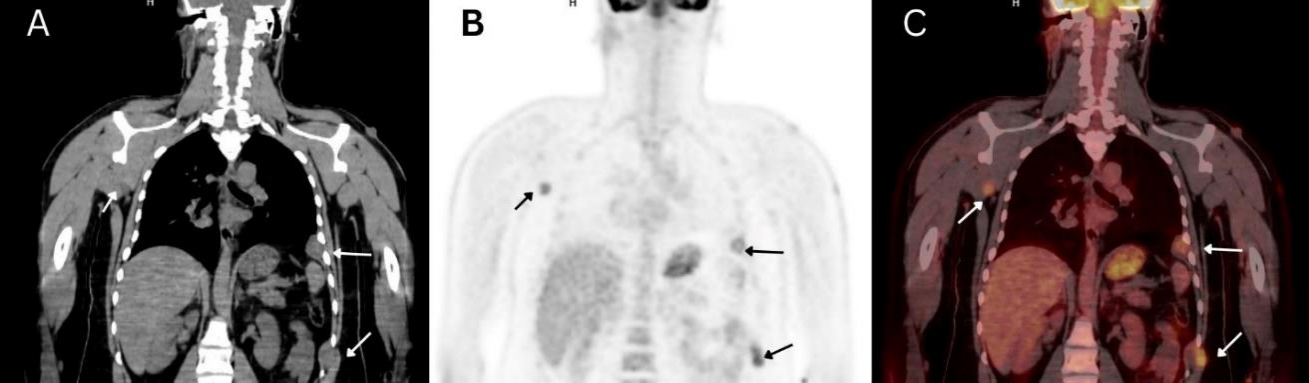
Coronal CT (**A**), PET (**B**), and fused images (**C**) demonstrate hypermetabolic two left pleural and intercostal soft tissue masses, as well as, a soft tissue lesion in the right axillary

**Figure 4:**
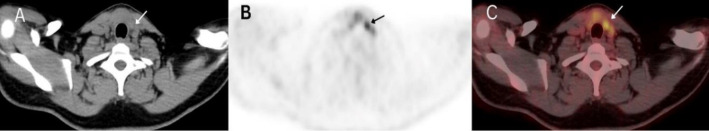
Axial CT (**A**), PET (**B**), and fused images (**C**) demonstrate hypermetabolic residual lesions in the bilateral thyroid bed, which is more prominent on the left side

 FDG PET/CT was ordered for better evaluation of the aforementioned lesions, and it showed hypermetabolic soft tissue lesions in the bilateral thyroid bed (SUV_max_: 6.0), left pleura, intercostal, and bilateral axillary regions, along with right inferior pelvic mass inseparable from the adjacent muscles (SUV_max_: 6.9) (Figures 2, 3, and 4). 

 Histopathological assessment of biopsied samples from the pleural and intercostal soft tissue masses confirmed the presence of neurofibromas with atypia. This led to the diagnosis of neurofibromatosis type 1 (NF1). A biopsy of the nodule on the left side of the thyroid revealed a malignant peripheral nerve sheath tumor that had not gone away, so a second neck exploration with residual tumor resection was done. The patient is currently well and under surveillance for the detection of any transformation of the known neurofibromas with atypia.

## Discussion

 MPNSTs are soft tissue sarcomas that are cancerous, locally aggressive, and have nerve sheath differentiation. They can start in a peripheral nerve or an existing benign peripheral nerve sheath tumor (BPNST) in people between the ages of 30 and 50. It can occur sporadically, but it is typically seen in association with genetic disorders such as neurofibromatosis type 1 (NF1), which is an autosomal dominant disorder caused by a mutation in the neurofibromin-1 gene on chromosome 17 (2). NF1 is diagnosed based on specific criteria such as café-au-lait macules, neurofibromas, freckles, optic pathway gliomas, lisch nodules (melanocytic hamartomas of the iris), bony lesions, or family history (3, 7). 

 Patients with NF1 have a risk of 8-13% for developing MPNST in their lives, compared to 0.001% lifetime incidents in the general population (8). Immunohistochemistry can help doctors figure out if someone has NF1-related MPNST. It mostly depends on whether or not Schwann cells have changed, which can be seen by testing for S100 protein or SOX10 expression (9).

 Thyroid involvement in MPNSTs is exceedingly rare, with fewer than 30 cases reported worldwide. Also, even though NF1 is a known high-risk factor for MPNSTs, no cases of thyroid involvement in NF1-associated MPNSTs have been reported. Instead, there have only been cases that look like thyroid tumors but don't have infiltration (10, 11). To our knowledge, this report presents the first case of thyroid involvement in an NF1-associated MPNST and the role of FDG PET/CT in its evaluation.

 Up to 50/% of all MPNSTs are associated with NF1 and they tend to occur two decades earlier than sporadic cases (3). Our patient was a 33-year-old male who was not diagnosed with NF1 prior to his presentation with MPNST, but his relatively young age raised the possibility that his case was not sporadic. Clinical examination supported the treating team’s suspicions by exposing widespread neuro-fibromas and café au lait spots. Finally, correlation with histopathology revealed that the patient had focally positive S100 expression, which is only present in the case of Schwann cell differentiation. While our patient’s CT scan was useful in the detection of other soft tissue lesions, it did little to differentiate malignant lesions from benign ones, which are common in NF1, so FDG PET/CT was ordered to aid in discerning benign neurofibromas caused by NF1 from MPNST through metabolic activity.

 A review by Treglia et al revealed that PET was able to detect malignant lesions with a sensitivity ranging between 89% and 100% and a specificity between 72% and 100% (12). 

 Moreover, unlike CT and MRI which lack precise discrimination between benign and malignant lesions, FDG PET/CT offers metabolic information through standardized uptake values (SUVs) in addition to anatomic evaluation. PET/CT was proven superior to conventional CT in detecting locally recurrent disease with a sensitivity of 100% compared to 86% with conventional CT (13) and higher FDG accumulation was noted in MPNST compared to benign lesions with a mean SUV_max_ of 7.5 vs. 1.9, respectively, and an accuracy of 83.5% (14) Multiple trials in the literature found that the optimum SUV_max_ threshold for differentiation likely fell somewhere between 3.0 and 4.0, with many using 3.5 as a cutoff value to differentiate BPNSTs and MPSNTs in their studies. However, Broski et al. found the higher cutoff of 4.3 to have a higher sensitivity in determining the benign nature of the lesion. However, they concurred with the literature using 3.5 as the cutoff value as it would avoid the risk of missing an early diagnosis (12-17). Broski et al. also found that SUV_max_ values between 4.3 and 8.1 had a low number of false-positive results on MRI so they recommend that as the next step in management in patients undergoing evaluation with biopsy being the test of choice for any lesion with SUV_max_ above 8.1 (16).

 In our case, FDG PET/CT was very helpful in figuring out how bad the disease was and how it affected metabolism. It also helped us tell the difference between benign neurofibromas and MPNST, which was not possible with regular CT and MRI scans. The left thyroid bed nodule's high SUV_max_ value of 6.0 meant that the disease was still present or recurred. This was confirmed by biopsy, and the patient had to have a second neck exploration to remove the tumor. The high SUV_max_ also raised concerns about the aggressive nature of the MPNST, highlighting the pivotal role of FDG PET/CT in providing valuable insights regarding prognosis and the determination of management pathways. 

 Stephen M. Broski et al. offered tailored recommendations for the management of peripheral nerve sheath tumors (PNSTs) based on their standardized uptake values (SUV_max_). 

 They advised that PNSTs displaying a SUV_max _of less than 4.3 could confidently be approached as BPNSTs, with consideration for surgical resection in cases of symptomatic lesions. An MRI evaluation is required for lesions exhibiting a SUV_max_ between 4.3 and 8.1 due to their low false-positive outcomes. When encountering PNSTs with a SUV_max_ surpassing 8.1, a biopsy is recommended, targeting the region of highest FDG avidity within the mass (16).

 Zoë Y.G.J. van Lierop et, al. revealed that FDG PET/CT is useful in detecting lesions other than nervous system tumors in patients with NF1, as there were 11 cases with thyroid lesions out of 69 NF1 patients, were diagnosed with either primary thyroid cancer, metastases, or adenomatous goiter disease, with SUV_max_ ranging 2.7±3.5, as none of them were diagnosed with thyroid nerve sheath tumor (18). Also, Yoshihiro Nishida et al. found 5 cases of thyroid incidentaloma (adenoma, thyroiditis) in 36 NF1 patients (19).

 In conclusion, this rare case of a primary thyroid MPNST in a patient with NF1 emphasizes the diagnostic complexity and aggressiveness of MPNSTs, especially when associated with NF1. The association between MPNSTs and NF1 underscores the importance of vigilant monitoring and early detection. In addition, the case highlights the valuable role of FDG PET/CT in delineating the extent of the disease, distinguishing benign from malignant lesions, supporting treatment decisions, and improving patient care. Overall, this case highlights the need for a multidisciplinary approach and heightened awareness of the effective management of MPNSTs associated with NF1, which can improve patient outcomes.
